# Zika Virus Outbreak, assisted reproduction patients and pregnancy

**DOI:** 10.5935/1518-0557.20180007

**Published:** 2018

**Authors:** Beuy Joob, Viroj Wiwanitkit

**Affiliations:** 1 Sanitation 1 Medical Academic Center, Bangkok Thailand; 2 Hainan Medical University, China

Dear editor, the publication on Zika virus (ZIKV) outbreak is very interesting. Borges *et al*. (2017) raised an interesting question: "*Should assisted reproduction patients avoid pregnancy*?" Borges *et al*. (2017) concluded that the "*ZIKV test before the onset of assisted reproduction treatment does not rule out the risk of the infection during pregnancy*" and "*In addition, although ZIKV infection risk is extremely high, the microcephaly risk due to ZIKV is not higher than the risk of miscarriage and birth defects due to other recognized pathogens*." We would like to share ideas and discuss this topic. First, we agree that the Zika virus test before pregnancy does not add any usefulness in prevention of the infection during pregnancy. The standard prevention against the Zika virus (mosquito prevention, sexual abstinence, avoidance of visiting the outbreak area, etc.) should be followed by all pregnant women. Second, we agree that the microcephaly risk due to ZIKV might not be high and severe. In our settings, a country in the tropical Southeast Asia are, the disease already exists but most infections are asymptomatic and there is no confirmed congenital case of microcephaly as a result of ZIKV in pregnant mother (Wiwanitkit & Wiwanitkit, 2016). Based on this from our settings, it is concluded that there is no need for either the general population or assisted reproduction patients to avoid pregnancy; nevertheless, the standard prevention methods must be followed by all.
